# Evaluation of *Russian sturgeon* (*Acipenser gueldenstaedtii*) Semen Quality and Semen Cryopreservation

**DOI:** 10.3390/ani12162153

**Published:** 2022-08-22

**Authors:** Violeta Igna, Ada Telea, Tiana Florea, Roxana Popp, Adrian Grozea

**Affiliations:** 1 Faculty of Veterinary Medicine, Banat’s University of Agricultural Sciences and Veterinary Medicine “King Michael I of Romania” from Timisoara, 300645 Timisoara, Romania; 2 Center for Gene and Cellular Therapies in the Treatment of Cancer—OncoGen, Clinical Emergency County Hospital “Pius Brinzeu” Timisoara, 300723 Timisoara, Romania; 3 Faculty of Bioengineering of Animal Resources, Banat’s University of Agricultural Sciences and Veterinary Medicine “King Michael I of Romania” from Timisoara, 300645 Timisoara, Romania

**Keywords:** Russian sturgeon, semen evaluation, cryopreservation, morphology, fertilization rate

## Abstract

**Simple Summary:**

The decline in sturgeon populations, including Russian sturgeons, has reached alarming levels, threatening these species with extinction. The requirements for the meat of these fish, caviar and other products used in the textile, medical and food industries are constant or even on the rise. Reproductive biotechnologies seem to be the main solution to these problems, but the many unknowns related to the particularities of the species still place them far from the efficiency reached in some species of domestic animals. The development of fish farms for sturgeons and the intensification of reproductive biotechnologies is the main way in which the natural habitats affected by depopulation can be repopulated and to respond to the consumption and industrial requirements of the population. Our purpose is to contribute with additional novel information on the cryopreservation of sturgeon spermatozoa, as well as the evaluation of semen quality with the aim of developing a cryopreservation technique suitable for commercial breeding facilities.

**Abstract:**

The alarming decline in sturgeon populations doubled by growing demands for sturgeon products, urge us to prevent the loss of these species. Fish stocking in natural habitats and developing fish farms are viable solutions, which can be successfully implemented with the help of reproductive biotechnologies. Despite the fact that semen cryopreservation is admittedly an important step for saving the Russian sturgeon, a reproducible standard method with good results has yet to be identified. Sperm quality assessment is essential for quantifying the impact of cryopreservation on spermatozoa. The purpose of our study was to provide additional information regarding semen cryopreservation and semen quality evaluation for the Russian sturgeon. Our study method is based on the use of two yolk-free extenders (with different cryoprotectants: DMSO and methanol) for freezing semen, using a simple freezing protocol. Parameters such as volume, concentration, motility, morphology and membrane integrity were evaluated. In conclusion, cryopreservation of Russian sturgeon spermatozoa using an extender containing methanol as cryoprotectant led to high egg fertilization percentages (72.67 ± 5.4%) even if the total motility values recorded for thawed semen were quite low (18–25%). Additionally, we identified two optimal stains for morphological studies and morphometry (Spermac stain kit and Trypan Blue Solution).

## 1. Introduction

The Russian sturgeon (*Acipenser gueldenstaedtii*) is one of the most widely distributed representatives of the Acipenser genus—it inhabits the basins of the Black, Caspian and Azov seas, with separate local stocks occurring in the large rivers entering these seas. The primary spawning river is the Volga and its tributaries. From the Black Sea, the sturgeon migrates for spawning to the Danube [1]. Russian sturgeon is the largest Danubian species of the genus Acipenser, and was the most widely distributed anadromous species in the Danube River [2] but now it is critically threatened with extinction. Drastic declines in sturgeon natural populations have led to sturgeons classifying as an endangered breed. The Russian sturgeon is currently included in the IUCN Red Data List [3]. The main causes and direct threats to sturgeon survival in the Danube River are overfishing, natural habitat destruction, pollution, and disruption of sturgeon migration routes. In addition to the legislative and financial measures taken in an attempt to fight off this ongoing natural disaster, sturgeon aquaculture has gained importance over the past years [4,5]. In nature, seasonal factors, such as water temperature and photoperiod play the main role in the annual growth response and maturation cycle of sturgeons. The reproductive cycle is regulated in natural conditions by external environmental factors that activate the reproductive axis. The lack of environmental stimulation in farm conditions means that the reared fish do not feel the conditions required for natural spawning. Therefore, for the farmed Russian sturgeon, it is generally essential to induce reproduction by manipulation of the endocrine system with exogenous hormones [6] and to collect the sperm and eggs from the broodfish for artificial reproduction [1].

In sturgeons, spermatozoa are produced in the testes then released into the sperm ducts during spawning season [7]. Sturgeon testes possess no externally opening sperm ducts. The efferent ducts coming from the testes are in direct contact with the kidneys. From the urogenital junctions, on their way to the external environment, the spermatozoa pass through Bowman’s capsules and tubules of the nephrons involved, then through the urinary collecting ducts, the wolffian duct and finally the sinus urogenitalis [8]. Therefore, it is believed that sperm becomes mixed with urine before being released or stripped from the genital papilla, which leads to a decrease in osmolality of the seminal plasma. Nevertheless, urine does not induce the activation of spermatozoa motility probably due to its potassium ions (K^+^) concentration. Research is needed to clarify the influence of this mixing process on spermatozoa motility, fertilizing ability and cryoresistance [9]. The mixing of sperm with excretory products has been found to be a requisite for the capacity to be activated (maturation step) instead of being deleterious [10]. Osmolality of the seminal plasma is a key factor to maintain spermatozoa in the quiescent state and K^+^ prevents spermatozoa motility. Once the spermatozoa are released during spawning, their motility is initiated in hypo-osmotic environments [11]. Sturgeon spermatozoa are immotile in the testes and in the seminal plasma; they become motile after being released into the aquatic environment [12]. Spermatozoa are immediately activated when they are transferred into the swimming environment which is usually fresh water or low salt concentration solutions [13]. Several parameters of the swimming medium, such as ion concentration (K^+^, Na^+^, Ca^2+^, Mg^2+^), osmotic level, pH and dilution rate, affect the motility of fish spermatozoa. Toth et al. [14] and Linhart et al. [15] reported the control of sperm motility by K^+^ concentration in sturgeon. The inhibitory K^+^ concentration for sperm activation has been reported to be between 0.1 mM and 2 mM in different sturgeon species. The biosensitivity of sperm towards Ca^2+^ and Na^+^ was reported by Toth et al. [14] and Cosson et al. [16]. Ca^2+^ and Na^+^ above 10 mM had a negative effect on sperm motility. The hypo-osmotic signal induces ionic exchange through the plasma membrane and triggers sperm activation through intracellular Ca^2+^ concentrations. A rise in Ca^2+^ also induces asymmetric flagellar beating in sturgeon sperm [17]. Prokopchuk et al. [18] has revealed that sturgeon spermatozoa may be activated by use of an unexpected signaling pathway, independent from ionic stimulation. This alternative regulation mechanism involves shock due to the osmotic difference (application of a hyperosmotic treatment immediately followed by dilution into a low-osmotic medium) and eliminates dependence on the previously described blocking effect of K^+^ ions. The optimal pH for sperm motility in Acipenseridae has been reported to be pH 8–9 in low dilution rate conditions 1:50 [14,19].

Among the sperm parameters, the morphology of Russian sturgeon spermatozoa has been little explored. To our knowledge, the only study was performed by Hatef et al. [20] who investigated spermatozoa morphology and fine structure in the Russian sturgeon, using scanning and transmission electron microscopy, although a wide variety of biological stains have been studied and are often used for human and different animal species for morphological assay in a bright microscopy. The stain and staining procedures used in humans or domestic animals are not always applicable to other species [21]. Usually, the stains are used to increase contrast, or to identify a particular region of spermatozoa. The lack of morphological studies performed in sturgeon species urges the initiation of feasible studies on this topic. The sperm membrane integrity assay, often referred to as the viability test, has been used extensively to evaluate sperm quality in men and domestic animals, using different methods. However, we did not identify any membrane integrity staining associated with light microscopy examination used in Russian sturgeon spermatozoa.

Recent research, focused on sturgeon breeding and reproduction, aimed at producing favorable effects on the conservation of wild sturgeon populations, on the repopulation of areas from which these species have disappeared, on the global demand for sturgeon meat and caviar, as well as on eco-toxicological evaluation of the aquatic environment, considering that the reproductive system of aquatic organisms, including spermatozoa, even cryopreserved spermatozoa, is most sensitive to the toxic effects of xenobiotics [22].

Reproductive biotechnologies in aquaculture have recently become more widespread, especially due to the threat of aquatic diversity. The development of effective artificial propagation techniques is crucial to expanding the aquaculture and to the development of preservation programs. In all species of farm animals, access to sperm through cryopreservation, regardless of time and location, has permitted the expansion of reproductive techniques which in turn have led to an acceleration of the genetic selection rate and conservation of the endangered species [23,24]. Selection of good quality milt is a necessary requirement for the cryopreservation of spermatozoa in endangered fish species including the Russian sturgeon [24].

Sperm quality assessment is one of the main pillars in aquaculture and reproductive biotechnology research. Several review articles are focused on methods for determination of sperm quality, sperm production indices, and chemical and biochemical compositions of the seminal plasma [25,26,27,28,29]. The range of optimal indicators should be defined according to the different species, sperm utility or reproductive strategy—such as artificial fertilization, cryopreservation, gene banking, or mass production [30]. Currently, there are no standardized methods or procedures for assessing the quality of sturgeon semen and no reference values for optimal indicators, related to different species. The sturgeon has become a popular species for aquaculture. One of the biggest challenges is to obtain viable eggs and spermatozoa at approximately the same time, from males and females, due to different individual responses to the hormonal stimulation treatment of spawning and spermiation.

The most common practice is short term preservation of undiluted semen at 4 °C, which can ensure the maintenance of viable sperm for a few days, prior to decline in fertility [31]. Refrigeration of undiluted semen at 4 °C is a simple and inexpensive procedure used in large-scale hatchery operations that bears the risk of contamination with water, making the semen non-viable. The cryopreservation of semen allows the sperm to be stored indefinitely, and this method has been recognized as the most appropriate way for gene banking aimed to preserve specific genetic diversity [32]. Relatively few studies have focused on the cryopreservation of Russian sturgeon spermatozoa [5,33,34,35] and there are still many unknown issues that need to be elucidated. Cryopreservation remains one of the most reliable methods for semen storage, and there are a few studies that report successful preservation of sturgeon semen, the majority of them using egg yolk in extenders, which carries the risk of contamination in commercial hatcheries [36].

The purpose of this paper is to provide additional information regarding Russian sturgeon (*Acipenser gueldenstaedtii*) semen quality evaluation and semen cryopreservation. We aimed to identify a reliable sperm staining method for spermatozoa morphology and morphometry analysis. In our study, we used yolk-free extenders to freeze the semen collected from *Acipenser gueldenstaedtii*, using a simple freezing protocol with the aim of developing a cryopreservation technique usable for commercial breeding facilities.

## 2. Materials and Methods

The experiments were carried out in the “Pădurea verde” (Farm no. 9) fish farm belonging to the Timișoara Didactical Station and in the Reproduction laboratory from Banat’s University of Agricultural Sciences and Veterinary Medicine from Timișoara, Romania, during the sturgeon breeding period and simultaneously with the artificial reproductive biotechnologies commonly applied on the farm. Spermiation induction procedures and techniques for semen collection in Russian sturgeons were those commonly used on the farm for sturgeon breeding. No additional treatment was applied to the animals included in the study. The farm is licensed for these activities.

The Russian sturgeon broodfish used in experiments were twelve-years-old, reared exclusively in a recirculating aquaculture system (RAS) in the above-mentioned fish farm. They were fed with SUPREME-10 dry food, specially formulated for sturgeons by Alltech-Coppens (Nettetal, Germany). The RAS used for broodfish rearing is installed in a 650-square-meter building, consisting of 4 tanks of 18 cubic meters each, and a water treatment unit with all the necessary equipment (drum filter, moving bed biofilter, air blower, pumping system, etc.). The water flow was adjusted to about 35 cubic meters per hour and tank, and the fresh water used for replacing 10 to 20% of the water from the RAS per day comes from a well. The water temperature fluctuates throughout the year between 9 °C and 23 °C in winter and summer, respectively. The reproduction of the sturgeon starts in early spring when the water temperature is maintained above 14 °C for 2 weeks.

The study included two experiments: evaluation of Russian sturgeon semen quality (experiment 1) and semen cryopreservation in the same species (experiment 2).

### 2.1. Experiment 1: Evaluation of *Russian sturgeon* Semen Quality

#### 2.1.1. Spermiation Induction and Semen Collection

Semen was collected during the breeding season from five different males, all 12 years old. Spermiation was induced using Luteinizing hormone-releasing hormone agonists (LH-RH, SIGMA-ALDRICH L4513), administered intramuscularly in a 20 μg kg^−1^ body weight dose, 24–30 h before the estimated time for semen collection [37]. All animal manipulation procedures were performed under clove oil anesthesia (0.5 mL L^−1^ for 5 min), and no animals were harmed or killed during these experiments. A plastic catheter was inserted through the urogenital orifice. An external abdominal massage in the area corresponding to the projection of the gonads was performed, in cranio-caudal direction. The semen was collected sequentially, and the different semen fractions were collected separately, in sterile 50 mL graded plastic containers. Immediately after collection, the semen samples were stored at 4–8 °C and transported to the laboratory for analysis and cryopreservation.

#### 2.1.2. Semen Quality Evaluation

Semen samples were assessed at the Reproduction laboratory. During their transportation from the fish farm to the laboratory, the samples were stored at 0–4 °C temperature, for approximately 1–2 h, in an ultra-dense styrofoam container, specially designed for the transportation of chilled semen (Minitube, Germany). In the laboratory, the samples were immediately removed from the transportation container and stored, during analysis, at 15–17 °C. The activation media and staining solutions were stored at the same temperature.

The volume of each sample was determined by directly reading the graded glass. The total volume of semen collected from each male was determined by adding up the values of all samples containing semen fractions from the same male.

The first stage of quality assessment was to check for semen contamination with water from the tanks, by microscopic examination of 10 µL of semen for each semen sample. The samples with more than 2% motile spermatozoa and the samples that did not activate were considered contaminated and were eliminated from the study.

The concentration (the number of spermatozoa per volume unit) and motility parameters were determined using the computer-assisted sperm analysis (CASA) system (IVOS, versions 12.3, Hamilton Thorne Bioscience, Beverly, MA, USA). The analysis of spermatozoa motility included the determination of total motility (TM%), average path velocity (VAP µm s^−1^) and total duration of movement (min). To activate the spermatozoa, 2 µL of semen were diluted with 50 µL of water from the fish tank (activation medium) and, within a maximum of 10 s after activation, the analysis of the motility parameters was performed. To perform computer-assisted analysis, a Leja counting chamber (IMV Technologies) was positioned on the heated plate of the integrated CASA system microscope, 3 µL of the semen-activator mixture was placed in one of the compartments of the Leja chamber and the computerized assay was initiated. The duration of maintaining the spermatozoa motility was monitored through microscopy (Olympus BX51, Olympus, Tokyo, Japan), from the moment the movement was initiated until it stopped. Spermatozoa morphology was assessed by microscopic examination after staining. A preliminary study was carried out prior to the morphological study in order to identify the optimal staining variant for Russian sturgeon spermatozoa, both for the morphological assay and for morphometry. Thus, four stains were selected: Diff-Quik (Medion Diagnistics AG, Düdingen, Switzerland), Spermac (Minitübe, Tiefenbach, Germany), Eosin G (Minitübe, Tiefenbach Germany) and Trypan Blue (Minitübe, Tiefenbach Germany). The selection of the four sperm staining methods was based on several criteria: simple and fast methods with previous successful application in other animal species or humans; intended for the morphological study of sperm; stained spermatozoa suitable for evaluation in bright microscopy; inclusion of two types of stains—with and without fixative solution.

The selected spermatozoa staining protocols were:

Diff Quik staining: the standard method recommended by the World Health Organization in the WHO laboratory manual 2010 [38]. The air-dried semen smear was immersed in the fixator solution of the kit for 15 s, after which sequential immersion was performed in solution I for 10 s and in solution II for 5 s, followed by washing with tap water, 10–15 immersions.

Spermac is a diagnostic staining kit for the morphology evaluation of sperm cells in all domestic mammals. The semen smear was fixated by immersion into the fixative solution for 5 min, followed by air-drying at 37 °C for 15 min. The proper staining was achieved by successively immersing the smear in the three stains—A, B and C for 1 min each. Each staining was followed by washing in tap water. Finally, the smear was allowed to air dry. In mammals, the head of spermatozoa appears red-colored, the acrosome, centerpiece and tail are green and the equatorial zone is pale green. The identification of damage to head and acrosome, as well as tail abnormalities are easily detected.

Eosin is a simple, rapid and effective stain. Two variants was performed:

Variant 1: the Eosin G solution was added over the dry semen smear and kept for 2 min when it was washed by successively immersing the slide in tap water, to remove the dye;

Variant 2: a drop of semen of 10 µL was placed at the end of a slide, over which 10 µL Eosin G solution was added and slightly homogenized. A smear of the mixture of sperm + eosin was prepared and left for 2–3 min at room temperature, then washed by successively immersing the slide in tap water;

Trypan Blue stain: 10 µL of semen was placed at the end of a slide, over which a drop of Trypan blue was added and homogenized slightly. A smear was prepared from the sperm + stain mixture and left at room temperature for 2 min, then washed by successively immersing it in tap water.

To assess spermatozoa morphology status in semen samples, the stained spermatozoa were examined in bright field microscopy, at 400–1000× magnification. Two hundred spermatozoa per sample were evaluated and the percentage of morphological abnormalities were expressed.

Spermatozoa membrane integrity evaluation was performed using the Eosin-Nigrosin staining method and the sperm viability kit SYBR-14/PI (Invitrogen, Molecular Probes, Leiden, Netherlands).

The morphometry assays of *Russian sturgeon* spermatozoa were carried out on semen smears stained using the best staining variant resulting from this study, namely the staining method which provided a clear visualization of the cell outline and tail. One hundred and fifty spermatozoa, namely 30 spermatozoa for each male, were measured in order to obtain the following morphological parameters: spermatozoa head length; spermatozoa head width (maximum); spermatozoa tail length and total length. Stained spermatozoa morphometry was performed by using bright field microscopy (Olympus BX51 microscope) with ×400 and ×1000 magnification. Pictures were taken using a digital color camera (Color View II Olympus) and the previously mentioned measurements were performed using the cell analysis software (cell F Imaging Software for Life Science Microscopy Olympus, Soft Imaging Solution, Munster, Germany).

Eosin-Nigrosin is a staining technique that assesses the vitality of a sperm sample and is recommended by the WHO for human semen analysis. Nigrosin increases the contrast between the background and sperm heads, making sperm easier to visualize. Eosin-Nigrosin staining procedure from this study used 2 μL semen and 4 μL of Eosin which were mixed gently followed by a 15-second waiting time. The next step was the addition of 6 μL Nigrosin, mixed gently and proceeded to making a smear. The smear must be air-dried and the sample can be examined using bright-field microscopy.

SYBR-14/PI staining procedure: the sperm sample was diluted using a saline HEPES buffer solution in a 1:10 ratio. An aqueous solution was prepared using the SYBR-14 stain from the solution on stock (dilution ratio 1:50). Five μL of SYBR-14 staining solution were added over 1 mL of diluted sperm. The sample was stored in an incubator for 5–10 min, at 36 °C. A measure of 5 μL of propidium iodide was added to the sample and then stored in the incubator for another 5–10 min. The sample was examined using green filter cube fluorescence microscopy (Olympus). The peak excitation and emission wavelengths for propidium iodide are 530 nm and 617 nm and for SYBR 14, 489 nm and 516 nm.

The stained semen samples were analyzed in bright or fluorescent microscopy, according to the stain used. Two hundred spermatozoa were randomly selected for each sample and spermatozoa with damage to the structural integrity of the membrane (dead spermatozoa) and normal spermatozoa (live spermatozoa) were identified. The percentage of spermatozoa with damaged membranes was expressed.

### 2.2. Experiment 2: Cryopreservation of *Russian sturgeon* Semen

#### 2.2.1. Preliminary Study for Testing the Activation Media

A preliminary study related to Russian sturgeon sperm activation was made in order to improve the post-thawing motility parameters of cryopreserved semen. The activation studies were performed on semen samples from three of the five of the males initially included in the previously described semen quality assessment experiment. Due to low values of the average path velocity parameters (VAP µm s^−1^) resulting after sperm activation using fish-tank water, two of the males were excluded from both the study regarding various sperm motility activation media and from the sperm cryopreservation studies.

Three sperm fractions were collected from each male and activated using the activation media included in the study; the results are presented in Table 1.

The activation was performed as previously described. Total motility (%), progressive motility (%) and average path velocity (µm s^−1^) of the spermatozoa were analyzed within 10 s after adding the sperm to the activation medium, with the Animal motility software of CASA system (IVOS HTB, version 12.3 Hamilton Thorne Biosciences, Beverly, MA, USA).

#### 2.2.2. Semen Cryopreservation

Sperm from three Russian sturgeons and two extenders were included in the study. Immediately after sperm collection, according to the procedure described in Experiment 1, the following sperm parameters were determined, using the CASA system: semen concentration, total motility and velocity. The two employed extenders were: E1-50 mM NaCl, 5 mM KCl, 10 mM Tris, 10% DMSO [40] and E2-30 mM Sucrose, 1 mM KCl, 25 mM, Tris, 10% Methanol [17].

Semen cryopreservation included the following steps: mixing the semen with the extender (1:1, *v/v*); filling and sealing 0.5 mL straws (ultrasonic closure); equilibration for 5 min at room temperature; pre-freezing in liquid nitrogen vapor, by placing straws horizontally, at a 4 cm distance above the liquid nitrogen, on floating rack in Styrofoam box, for 10 min; immersion in liquid nitrogen; storage of cryopreserved semen in storage containers (MVS SC 20/20), until used; thawing cryopreserved sperm by immersion in water at 35 °C for 10 s, before using it to fertilize eggs. Evaluation of the impact of cryopreservation on spermatozoa was performed by assessing spermatozoa post-thaw motility and egg fertilization.

#### 2.2.3. Egg Fertilization

A sterlet (*Acipenser ruthenus*) female was stimulated for the final maturation of sex cells by intramuscular solution, 24 to 30 h before the eggs were stripped. The eggs were collected in a clean and dry plastic container. The ovarian fluid was removed and the eggs were fertilized by means of semidry technique applied for sturgeons in hatcheries [42]. A quantity of 10 g of eggs was distributed in plastic dishes for each experimental variant in triplicate and thawed semen was added, in a ratio of 10 mL of sperm/1 kg of eggs. Before the semen was placed over the eggs, it was thawed and mixed with an activator medium. After 3 min of keeping the sperm together with the eggs, the egg stickiness was eliminated with milk/talc mixture (0.5 kg of talc in 1 L of milk diluted with 7 L of water) [43]. The eggs were incubated at temperatures of 17–18 °C. Results’ evaluation was performed 12 h after artificial fertilization, by microscopy. Identification of fertilized and unfertilized eggs was performed on 30 eggs from each sample (90 eggs per variant) and the fertilization rate was calculated.

#### 2.2.4. Statistical Analysis

All laboratory data are presented as mean ± standard deviation (SD). The results were statistically analyzed using the factorial ANOVA followed by the Fisher and Mann–Whitney tests, using Statistica Software version 8.0.360.0, StatSoft Inc, Tulsa, OK, USA. *p* values less than 0.05 were considered significant.

## 3. Results

### 3.1. Semen Quality Evaluation

The results of the Russian sturgeon semen evaluation are shown in Table 2. The average volume of sperm collected from the five Russian sturgeons taken into the study was 57.1 ± 35 mL, with individual variations ranging from 22.8 to 100 mL. Sperm concentration ranged from 2211.5 × 10^6^ to 5779.1 × 10^6^ (2.2–5.7 × 10^9^) spermatozoa mL^−1^. The results of the computer analysis on the percentage of spermatozoa showing movement and their velocity are shown in Table 2.

Monitoring of spermatozoa movement duration, from the moment of activation using the activation medium, to the onset of immobility, revealed an average duration of individual movement persistence between 2 and 4 min. Mass movements of spermatozoa, similar to sperm waves of ruminant sperm, are exhausted much faster, and can be observed in the microscopic field for about 30 to 60 s.

**Figure 1:**
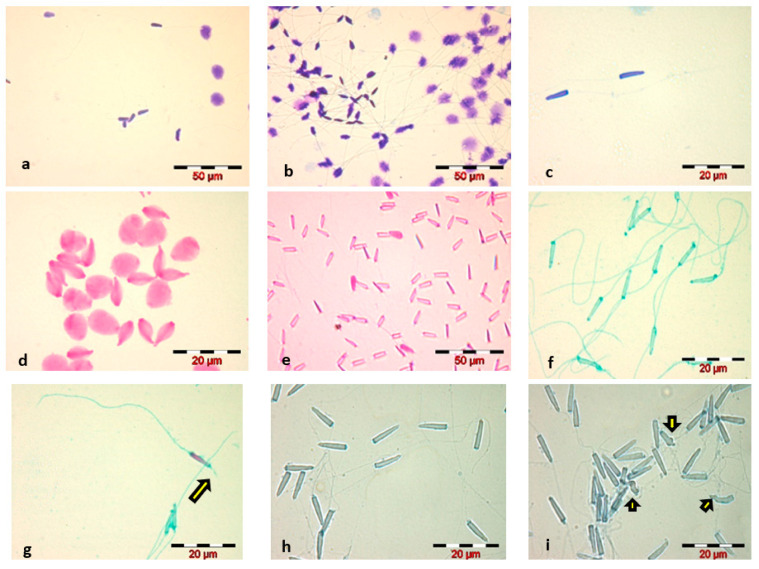
Microscopic images (×400, ×1000)—Russian sturgeon sperm smears stained using different methods: (**a**) Diff-Quik–WHO; (**b**) Diff-Quik–1 min.; (**c**) Diff-Quik–5 s; (**d**) Eosin G according to two types of stain-exposure time: 2 min and (**e**) 5 s; (**f**,**g**) Spermac—the yellow arrow indicates the acrosome reaction (acrosomal filament); (**h**,**i**) Trypan Blue—the yellow arrows indicates spermatozoa head abnormalities.

Staining of Russian sturgeon spermatozoa with the four stains selected for the present study revealed the following:

- ***Diff-Quik staining***, WHO 2010 method, caused swelling of the sperm head and did not highlight the sperm tail—Figure 1a;
  Increasing the staining time to 1 min, for each component of the Diff-Quik kit, slightly stained the tail, but caused the sperm head to swell to a significant extent—Figure 1b;
  Reducing the immersion time to 5 s, for each of the three components of the Diff-Quik kit, resulted in good staining of the sperm head and avoidance of swelling, but very poor staining of the sperm tail—Figure 1c;
- ***Eosin staining***, in both variants, led to spermatozoa head swelling and faded spermatozoa tail staining—Figure 1d;
  Decreasing the staining time with Eosin to 5 s eliminated the phenomenon of sperm head swelling that occurred in the classical staining variant, but we considered the staining of spermatozoa inadequate for a morphological and morphometric evaluation of the sperm cell due to poor visualization of the head outline and very poor staining of the spermatozoa tail—Figure 1e;
- ***Spermac staining***, carried out according to the manufacturer’s instructions, resulted in good staining of the Russian sturgeon spermatozoa, with a clear highlighting of the spermatozoa morphological elements—Figure 1f; and even a marked highlighting of the acrosome reaction—Figure 1g.
- ***Trypan Blue staining*** showed a proper visualization of the spermatozoa head—Figure 1h.

Comparative analysis of the four stains used for Russian sturgeon sperm revealed that the Spermac staining kit and Trypan Blue were the most suitable methods for staining the spermatozoa of this species, providing an optimal visualization for morphological and morphometric analysis of spermatozoa.

The morphological analysis of the spermatozoa revealed the presence of abnormal sperm forms, the main identified abnormalities being located in the head—amorphous head—Figure 1i, in the intermediate piece—head detached from the tail, and in the tail—broken, curled, coiled, short, absent. The average percentage of spermatozoa with morphological abnormalities, calculated for the five analyzed sperm samples, was 9 ± 2.28%.

Spermatozoa measurement was performed on semen smears stained with Spermac. The results of the spermatozoa morphometric analysis are shown in Table 3.

**Figure 2:**
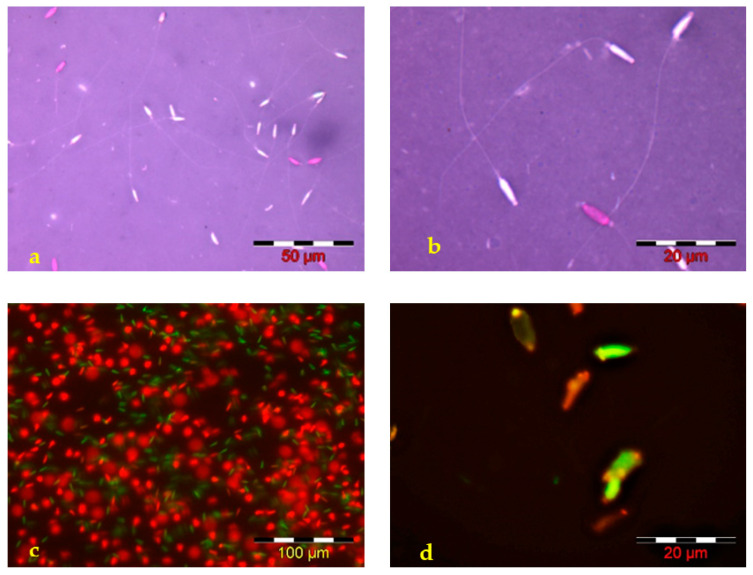
Microscopic images—Russian sturgeon sperm smears stained with (**a**,**b**) Eosin-Nigrosin (spermatozoa with an affected membrane stained in pink and spermatozoa with complete membrane integrity—not stained) and (**c**,**d**) with SYBR 14—PI end examined in fluorescence microscopy—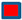 dead sperm cells, 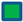 live sperm cells.

Analysis of the structural integrity of Russian sturgeon sperm membranes, performed using the two specific stains—Eosin-Nigrosin—Figure 2a,b and SYBR 14-PI—Figure 2c,d, revealed the presence of 17.60 ± 3.7% (range between 13–21%) spermatozoa with damaged membranes in the examined sperm samples. Both employed stains are suitable for the analysis of Russian sturgeon spermatozoa structural membrane integrity.

### 3.2. Cryopreservation of *Russian sturgeon* Semen

#### 3.2.1. Testing the Activation Media

The motility parameters according to the different semen activation media and different males are summarized in Table 4. The data showed very high variability of the studied indicators in the three males, depending on the activation media. These data corroborated with the ANOVA test indicated that both the male and the activation media had a significant effect (*p* ≤ 0.05) on total motility, progressive motility and velocity of spermatozoa.

The data from Table 4 indicated significant differences (*p* ≤ 0.05) between male 1 vs. male 2 and 3 with respect to total motility of the spermatozoa activated with different activation media. Significant differences (*p* ≤ 0.05) were also observed at path velocity of the spermatozoa activated with different activation media, between male 1 and male 3.

The data from Table 5 indicate few significant differences (*p* ≤ 0.05) between the activation media. Almost all activation media was suitable to activate spermatozoa motility, but only AM 3, activating in a significant, lower percent (54.44 ± 25.68%) the total motility of the spermatozoa, comparing with the other activation media used in our experiments. Corroborating these data with the other parameters, AM0, AM2 and AM4 showed the best results for spermatozoa activation.

#### 3.2.2. Semen Cryopreservation

The values of the semen parameters recorded in Russian sturgeons immediately after semen collection (volume), and after the arrival of the samples in the laboratory (concentration, total mobility and speed) are shown in Table 6.

**Figure 3:**
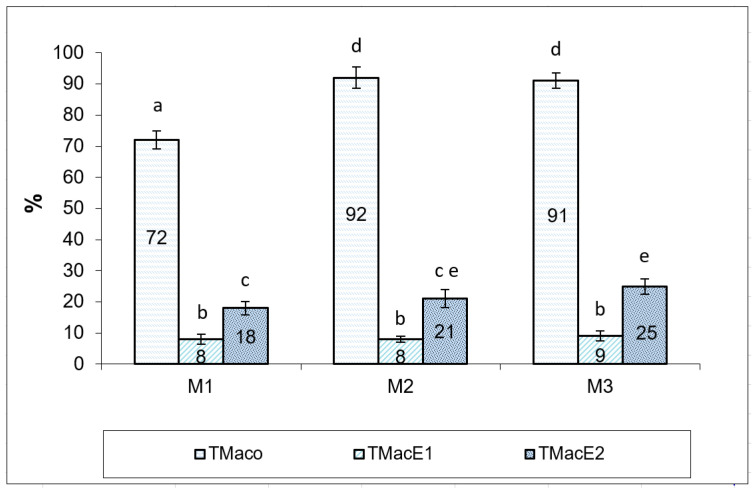
Spermatozoa total motility (%), registered in semen samples from the three Russian sturgeon males (M1, M2, M3), before and after cryopreservation with both extenders (E1 and E2). TMaco—total motility after collection; TMacE1—total motility after cryopreservation with extender 1; TMacE2—total motility after cryopreservation with extender 2. Different letters indicate significant differences (*p* ≤ 0.01).

The impact of cryopreservation on the motility of Russian sturgeon spermatozoa, in both experimental variants (cryopreservation media E1 and E2), are presented in Figure 3. Cryopreservation of Russian sturgeon semen has a significant, negative impact on sperm motility. The average percentage of total motility after thawing was 8.33 ± 0.47%, with limits between 8–9% for semen extended with E1 [40] and 21.33 ± 2.87%, with limits between 18–25% in semen extended with E2.

#### 3.2.3. Egg Fertilization

**Figure 4:**
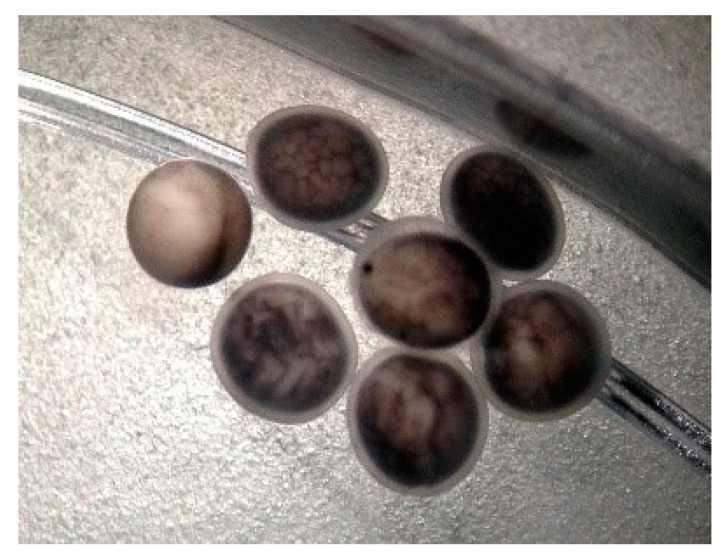
First stages of cleavage, 12 h after artificial insemination of sterlet eggs with cryopreserved Russian sturgeon semen: fertilized eggs with normal development, fertilized eggs with blastomere inhibition and unfertilized eggs.

Examination of the eggs at 12 h after artificial insemination, with the stereomicroscope, revealed the existence of: fertilized eggs in different stages of embryonic development, fertilized eggs with a disrupted cleavage process and unfertilized eggs—Figure 4.

**Figure 5:**
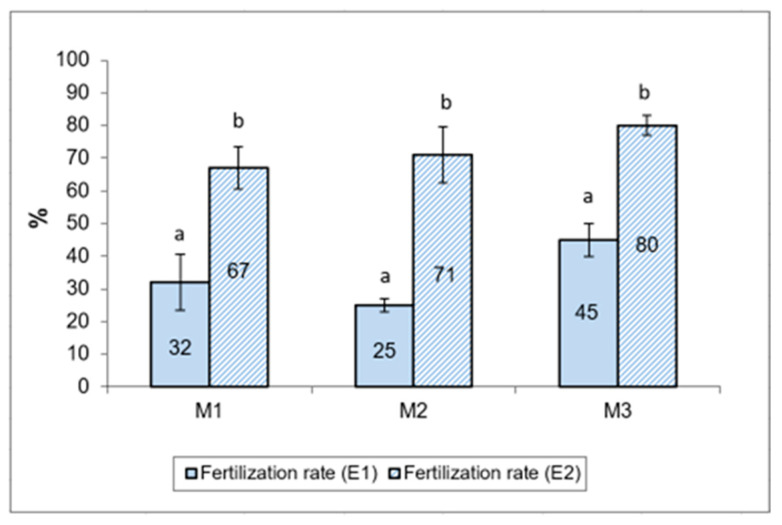
Sterlet eggs fertilization percentage, fertilized by cryopreserved semen of Russian sturgeon using the E1 and E2 extenders. Different letters indicate significant differences (*p* ≤ 0.01).

The fertilization results for sterlet (*Acipenser rhutenus)* eggs with Russian sturgeon (**Acipenser gueldenstaedtii*)* semen, cryopreserved with two different extenders (E1 and E2), are presented as percentages of fertilized eggs in Figure 5. The mean fertilization rate was higher in samples using cryopreserved semen with extender E2, using methanol as a cryoprotective agent (72.67 ± 5.44%), compared to samples using sperm preserved with extender E1, and using DMSO as cryoprotective agent (37.50 ± 5.50%).

## 4. Discussion

The purpose of our study was to provide additional information regarding semen cryopreservation and semen quality evaluation of the Russian sturgeon, given the absence of standardized methods or procedures for cryopreservation and assessment of semen on this species. The evaluation of semen quality is the key step in studies directed towards the influence of some factors on spermatogenesis and sperm-related parameters as well as studies focusing on certain compounds or technical parameters used in sperm cryopreservation, such as: sperm extenders, dilution rates, balancing time, packaging, cooling rates and thawing parameters, spermatozoa motility activation.

The mean semen volume value collected from the Russian sturgeons was lower than that reported by Halimi et al. [24], 86.3 ± 8.1 mL; however, it can be considered similar, given that in our study, one of the males (M2) gave out a smaller amount of semen during collection compared to the other males. A comparison of the results from the two studies reveals the possible influence of different factors on the volume of sperm collected: habitat (fish farm with recirculating aquaculture system versus southern part of the Caspian Sea), compounds used for spermiation induction (luteinizing hormone-releasing hormone agonists versus pituitary preparation PP) and other factors such as timing of sperm collection in relation to spawning period, handling techniques, restraint or anesthesia of males and method of sperm collection.

The average sperm concentration was 4216.78 × 10^6^ ± 1202.09, ranging between 2870.2 × 10^6^ (male M3) and 5779.1 × 10^6^ (male M1). The mean concentration value of 3.9 × 10^9^ spermatozoa/mL was well above the mean concentration reported by Li et al. [44] for a group of four males aged 10 years and with a body weight of 10–11 kg (0.19 × 10^9^ spermatozoa/mL). These differences could be due to the spermiation induction product and procedure (LHRH analogue versus carp pituitary extract), concentration determination method (computerized CASA analysis versus hemocytometric method using the Bürker chamber cell counting formula) and husbandry technology employed for the males. Such within-species differences have also been obtained by other researchers and explained by the different origin of broodfish, collection period and sperm collection methods. Semen volume and concentration generally vary in males of different animal species, even if they are of the same age and live in an environment with similar technological conditions. Sturgeon semen samples from the present study support the influence of the individual factor on semen parameters.

The mean of the total motility—TM% (74.4 ± 11.9) was similar to that recorded by Halimi et al. [24] (69.6 ± 3.5%). The mean values of sperm mobility persistence of Russian sturgeon spermatozoa recorded in this study (2–4 min) were similar to those reported by Drabkina cit. by Aramli et al. [28] for Russian sturgeon (3–6 min).

The morphological study of cells often requires staining of the biological sample. The stains are used to increase contrast, or to identify a particular region of spermatozoa [21], although the staining methods may cause a slight change in the size measurement values as the fixatives may cause the cells to shrink slightly. Differences in human spermatozoa head size values (length and width) were observed when two different staining methods were used: Papanicolau and Diff-Quik [45]. Our preliminary study was conducted to identify the optimal staining variant for Russian sturgeon spermatozoa and started with the same issues encountered in sterlet (*Acipenser ruthenus*) spermatozoa [46]. In our study, Spermac and Trypan Blue were found to be suitable for staining Russian sturgeon spermatozoa, providing an optimal visualization for morphological and morphometric analysis in bright field microscopy. These could be the alternative to some analyses performed in electron microscopy. These techniques require specific and meticulous sample preparation. Individual microscopes are expensive and samples are often transferred to specific laboratories specializing in these techniques [21]. The identification and description of different types of abnormalities, as well as the development of criteria for the evaluation of sperm morphology are important steps for the standardization of the morphological analysis of Russian sturgeon, similar to those in humans or some species of domestic animals. The results of the spermatozoa morphometric analysis were similar to those reported by other authors [7,20]. The morphological study carried out on sperm samples stained with Spermac revealed the presence of acrosomal filaments in some spermatozoa. We consider that this observation could be the basis for new studies on staining methods. Further research should be focused on determining whether the Spermac stain kit is effective in highlighting the acrosome reaction in Russian sturgeon or whether there are other better alternatives for this purpose. In addition to electron microscopy techniques, the acrosome reaction in *Acipenser ruthenus* spermatozoa were observed under phase contrast microscopy at 400× [47] and 100× magnification [48,49]. Lahnsteiner et al. [40] used 4% formaldehyde to fix sperm samples for evaluating the acrosomal reaction.

The sperm membrane integrity assay, often referred to as viability test, has been used extensively to evaluate sperm quality in men and domestic animals, using different methods. There are some sperm viability kits, such as SYBR-14 and Propidium Iodide (PI), suitable for use with the flow cytometer and fluorescence microscope. Horokhovatskyi et al. [50] used the SYBR-14 and PI viability test for live/dead sperm ratio determination in sterlet (*Acipenser ruthenus*). Sperm cells were analyzed using a flow cytometer. Although demonstrated to be an accurate and relatively easy-to-use instrument, the flow cytometer is expensive and requires specialized training [21]. The membrane integrity analysis of Russian sturgeon spermatozoa by staining and bright field microscopy examination has not yet been reported. Our results recommend both stains to analyze membrane integrity: Eosin-Nigrosin in bright field microscopy and SYBR 14-PI in fluorescent microscopy.

Currently, cryopreservation has a detrimental effect on the structural and functional parameters of Russian sturgeon spermatozoa [5,34,35,51]. Many factors influence the success of cryopreservation, including the initial quality of semen, the composition of the cryomedium, the cryoprotectant, the dilution factor, the concentration of spermatozoa used for cryopreservation, speed of freezing and thawing, as well as physiological aspects of sperm, such as species specificity [52,53,54]. Protocols cause damage to cell structure and physiology, altering sperm functioning due to cryo-injuries during freezing and thawing [55]. A decrease in the percentage of spermatozoa motility as a result of the freeze-thawing process is a well-known phenomenon [17,56]. Our results showed a significant negative impact of cryopreservation on the mobility of Russian sturgeon spermatozoa, as described in other previous studies [34]. This impairment of motility has been studied in fish, revealing that cryopreservation has induced changes and/or damage to mitochondria [55]. A direct correlation exists between the mitochondrial membrane potential (transmembrane integrity, ∆Ψm) and the motility and fertilizing capacity of the cryopreserved spermatozoa [55].

One of the most important components of the extenders used in sturgeon semen cryopreservation is the cryoprotectant. The cryoprotectant agents are added to the sperm in order to increase the survival of spermatozoa during the freezing procedure [57]. Dimethyl-sulfoxide (DMSO) and methanol (MeOH) were each found to be effective cryoprotectants for sturgeon sperm [58,59,60]. However, the fertilization and hatching success achieved with DMSO as a cryoprotectant varied among species [56]. Data referring to the two cryoprotectants are however sometimes contradictory. Earlier works indicated that the cryopreservation of sturgeon spermatozoa using DMSO-sucrose extender resulted in the recovery of motile spermatozoa with basic motility characteristics similar to those of fresh semen. DMSO gave the best protective effect on Ponto-Caspian sturgeon sperm [57]. High post-thaw motility, but low or non-existent fertilization rates of sturgeon spermatozoa cryopreserved with the use of DMSO have been reported by other authors [13,40,56]. MeOH as a cryoprotectant secures both high values of sperm motility and velocity, although sometimes lower motility and fertilization rates than DMSO have been observed [40,59]. The usefulness of methanol for sperm cryopreservation has been confirmed for the sterlet (*A. ruthenus*). Lahnsteiner et al. [40] have recorded a significant reduction in the fertilizing ability of sterlet semen frozen with DMSO, while fertility rates with semen frozen with methanol were comparable to controls.

The results regarding the fertilization of eggs were somewhat unexpected, for both extenders used in the study, considering the low total motility of the thawed semen. The absence of strong relationships between sperm motility parameters and their fertilization ability after cryopreservation was also reported in sterlet [17]. In our study, low post-thaw sperm motility, particularly in samples where DMSO was used, was associated with slightly higher fertilization rates. We explain this somewhat unexpected result by the fact that in the case of in vitro fertilization, the spermatozoa are added over the eggs and an immediate and closer direct contact between the two gametes is ensured, compared to natural fertilization. The acrosome undergoes a change when the sperm contacts the egg or its surface components. This usually involves exocytosis of the acrosoma1 contents with the subsequent formation of an acrosomal filament or process. This protrusion of the acrosomal filament must occur in the micropyle of the egg for successful fertilization [47]. It is well established that the sperm acrosome is necessary for penetration of the spermatozoon into the egg, but its precise physiological role is still unknown [61]. Additional studies are needed to show which spermatozoa parameters are most closely correlated with fertilization in the case of in vitro fertilization. It is possible that the acrosome reaction rate or percentage of normal morphological spermatozoa with structural membrane integrity play the more important role as indicator of fertilization success. However, the data obtained by Psenicka et al. [48] strongly suggest that the reduced fertilization capability of the sterlet spermatozoa post-cryopreservation with DMSO is related to its harmful specific effect towards the acrosome.

The fertilization rates in our study are promising and should be considered as a starting point in other studies regarding cryopreservation. The optimum method of cryopreservation varies among fish [62]. The feasibility and effectiveness of each potential cryodiluent should be thoroughly investigated [63].

The different activation solutions selected and used in our study did not have the expected positive impact on the sperm motility parameters of Russian sturgeon. Since AM0 is water from the fish breeding tank, despite individual differences, from a practical and technological point of view, there are no real advantages in using a different activation solution. We consider that future research is needed in order to identify an activation medium capable of improving the main motility parameters of Russian sturgeon spermatozoa in both fresh and preserved semen for short-term or long-term.

## 5. Conclusions

Semen collected from farmed Russian sturgeons during the breeding season displayed individual variations of semen volume, concentration and motility parameters.

Russian sturgeon spermatozoa stained with Spermac stain kit or Trypan Blue Solution provided an optimal view of the cell outline and structural components, which is necessary in morphological and morphometric studies.

Cryopreservation of Russian sturgeon spermatozoa using an extender containing methanol as cryoprotectant (30 mM Sucrose, 1 mM KCl, 25 mM, Tris, 10% methanol), led to high egg fertilization rates (72.67 ± 5.44%) even if the total motility values recorded for thawed semen were quite low (18–25%).

## Figures and Tables

**Table 1:** Activation media (AM) used to activate fresh sturgeon spermatozoa.

Activation Media		References
AM0	water from tank	[9]
AM1	10 mM Tris, 20 mM NaCl, 2 mM CaCl_2_, pH 8.5	[39]
AM2	12.5 mM NaCl, pH 8.5	[40]
AM3	12.5 mM NaCl + 1 mM MgSO_4_	[40]
AM4	12.5 mM NaCl + 1 mM CaCl_2_	[40]
AM5	10 mM NaCl, 1 mM CaCl_2_, 10 mM Tris–HCl, pH 8.5	[41]

**Table 2:** Sperm parameters recorded in Russian sturgeon semen samples immediately after collection.

Male	Volume of Collected Semen (mL)	Concentration (nr.spz mL^−1^)	Total Number of Spermatozoa	Total Motility (TM%)	Velocity (VAP µm/s)
M1	22.8	4907.2 × 10^6^	111.88 × 10^9^	69	88.16
M2	26.7	2870.2 × 10^6^	76.63 × 10^9^	79	88.05
M3	49.0	2211.5 × 10^6^	108.36 × 10^9^	60	95.23
M4	100.0	5779.1 × 10^6^	577.9 × 10^9^	72	42.75
M5	87.0	4001.05 × 10^6^	410.64 × 10^9^	92	48.2
Mean ± SD	57.1 ± 35	3953.8 × 10^6^ ± 1453.6	257.08 × 10^9^ ± 224.8	74.4 ± 11.9	72.47 ± 24.8

**Table 3:** The values of the analyzed morphometric parameters for Russian sturgeon spermatozoa stained with Spermac.

Parameter (Measuring Unit)	Measured Values (Mean ± Standard Deviation)	Values Cited in the Literature [20]
Head length (µm)	8.01 ± 0. 40	8.02 (1.18/AL + 6.84/NL) *
Head width (µm)	1.34 ± 0.20	1.48 (PNW) *
Tail length (µm)	53.91 ± 2.07	51.06 (1.64/ML + 49.4/FL) *
Total sperm cell length (µm)	61.88 ± 2.33	57.08

* AL = acrosome length, NL nucleus length, PNW posterior nucleus width, ML = midpiece length, FL = flagellar length.

**Table 4:** The motility parameters according to the different semen activation media at different males. Different letters of the mean ± SD on table columns indicate significant differences (*p* ≤ 0.05).

Male Sturgeon	Activation Media	Total Motility (TM) (% ± SD)	Progressive Motility (PM) (% ± SD)	Path Velocity (V) (µm s^−1^ ± SD)
M1	AM0	69.00 ± 14.00	12.33 ± 6.66	88.17 ± 31.39
	AM1	45.67 ± 17.79	10.00 ± 13.23	79.70 ± 44.64
	AM2	43.33 ± 11.93	12.67 ± 8.08	106.20 ± 10.71
	AM3	32.67 ± 9.02	11.33 ± 7.57	107.37 ± 39.89
	AM4	57.67 ± 22.12	17.00 ± 16.64	91.10 ± 11.97
	AM5	71.33 ± 18.01	16.00 ± 21.66	71.70 ± 23.59
	Mean ± SD	53.28 ± 19.74 ^a^	13.22 ± 11.60 ^a^	90.71 ± 28.48 ^a^
M2	AM0	78.67 ± 12.01	28.67 ± 2.52	88.07 ± 14.15
	AM1	75.33 ± 8.02	5.67 ± 2.52	56.17 ± 6.69
	AM2	81.00 ± 16.00	30.33 ± 4.04	92.60 ± 7.92
	AM3	61.00 ± 25.06	17.33 ± 2.08	78.00 ±20.35
	AM4	51.67 ± 2.52	11.67 ± 5.51	90.37 ± 7.64
	AM5	70.67 ± 9.07	5.33 ± 1.15	62.57 ± 1.75
	Mean ± SD	69.72 ± 15.88 ^b^	16.50 ± 10.70 ^a^	77.96 ± 17.34 ^a,b^
M3	AM0	60.33 ± 20.03	26.00 ± 12.00	95.23 ± 3.57
	AMI	60.00 ± 22.65	1.00 ± 1.00	41.30 ± 8.40
	AM2	91.33 ± 9.29	23.67 ± 13.01	69.67 ± 13.58
	AM3	69.67 ± 28.38	11.00 ± 6.00	61.83 ± 31.17
	AM4	63.33 ± 32.13	25.00 ± 11.53	86.80 ± 12.47
	AM5	51.67 ± 14.57	2.33 ± 2.08	51.33 ± 15.70
	Mean ± SD	66.10 ± 22.89 ^b^	14.83 ± 13.24 ^a^	67.69 ± 23.84 ^b^

**Table 5:** The motility parameters according to the different semen activation media. Different letters on table columns indicate significant differences (*p* ≤ 0.05).

Activation Media	Total Motility (TM) (% ± SD)	Progressive Motility (PM) (% ± SD)	Path Velocity (V) (µm s^−1^ ± SD)
AM0	69.33 ± 15.76 ^a,b^	22.33 ± 10.31 ^a^	90.49 ± 17.67 ^a^
AM1	60.33 ± 19.71 ^a,b^	5.56 ± 7.80 ^b^	59.06 ± 28.43 ^b^
AM2	71.89 ± 24.49 ^b^	22.22 ± 11.07 ^a^	89.49 ± 18.61 ^a^
AM3	54.44 ± 25.68 ^a,c^	13.22 ± 5.83 ^b,c,d^	82.40 ± 33.82 ^a^
AM4	57.56 ± 20.19 ^a,b^	17.89 ± 11.99 ^a,d^	89.42 ± 9.66 ^a^
AM5	64.56 ± 15.76 ^a,b^	7.89 ± 12.54 ^b^	61.87 ± 16.72 ^b^

**Table 6:** Sperm parameters recorded in Russian sturgeon semen samples immediately after collection.

Male	Volume of Collected Semen (mL)	Concentration (nr.spz mL^−1^)	Total Motility (%)	Velocity (VAP) (µm s^−1^)
M1	100	5779.1 × 10^6^	72	42.7
M2	87	4001.05 × 10^6^	92	48.2
M3	26.7	2870.2 × 10^6^	91	73.9
**Mean ± SD**	72.23 ± 31.93	4216.78 × 10^6^ ± 1202.09	85 ± 9.20	54.93 ± 13.60

## Data Availability

Not applicable.
